# Development and Validation of Protein Microarray Technology for Simultaneous Inflammatory Mediator Detection in Human Sera

**DOI:** 10.1155/2014/820304

**Published:** 2014-10-14

**Authors:** Senthooran Selvarajah, Ola H. Negm, Mohamed R. Hamed, Carolyn Tubby, Ian Todd, Patrick J. Tighe, Tim Harrison, Lucy C. Fairclough

**Affiliations:** ^1^School of Life Sciences, The University of Nottingham, A Floor West Block, Queen's Medical Centre, Nottingham NG7 2UH, UK; ^2^Roche Products Limited, 6 Falcon Way, Shire Park, Welwyn Garden City AL7 1TW, UK; ^3^Medical Microbiology and Immunology Department, Faculty of Medicine, Mansoura University, Mansoura 35516, Egypt; ^4^Nottingham Respiratory Research, University of Nottingham Clinical Sciences Building, City Hospital Campus, Nottingham NG5 1PB, UK

## Abstract

Biomarkers, including cytokines, can help in the diagnosis, prognosis, and prediction of treatment response across a wide range of disease settings. Consequently, the recent emergence of protein microarray technology, which is able to quantify a range of inflammatory mediators in a large number of samples simultaneously, has become highly desirable. However, the cost of commercial systems remains somewhat prohibitive. Here we show the development, validation, and implementation of an in-house microarray platform which enables the simultaneous quantitative analysis of multiple protein biomarkers. The accuracy and precision of the in-house microarray system were investigated according to the Food and Drug Administration (FDA) guidelines for pharmacokinetic assay validation. The assay fell within these limits for all but the very low-abundant cytokines, such as interleukin- (IL-) 10. Additionally, there were no significant differences between cytokine detection using our microarray system and the “gold standard” ELISA format. Crucially, future biomarker detection need not be limited to the 16 cytokines shown here but could be expanded as required. In conclusion, we detail a bespoke protein microarray system, utilizing well-validated ELISA reagents, that allows accurate, precise, and reproducible multiplexed biomarker quantification, comparable with commercial ELISA, and allowing customization beyond that of similar commercial microarrays.

## 1. Introduction

A biological marker, or “biomarker,” is a quantifiable characteristic that, when measured, may indicate normal biological processes, a pathogenic state, or a pharmacological response to a therapy [1]. As such, biomarker identification may help to diagnose disease, aid in the prognosis of disease, and/or help to predict treatment outcome. DNA, RNA, proteins, cytokines, and chemokines can all act as biomarkers, sources of which include local tissue, peripheral blood, and urine (reviewed in [2]).

Cytokine biomarkers play an important role in a vast range of diseases. These include but are not limited to interferon-*γ* (IFN-*γ*) and IFN-*γ*-induced protein 10 (IP-10) in* Mycobacterium tuberculosis*, which can aid in the diagnosis of disease, as well as vaccine-induced protection [3, 4]; CCL2 in HIV infection, which correlates with viral load [5]; tumour necrosis factor-*α* (TNF-*α*), interleukin (IL)-1*β*, and IL-6 in heart failure, which are associated with more severe disease and adverse outcome [6, 7]; and TNF-*α* and IL-6 in major depressive disorder, which correlate to poor antidepressant response [8, 9].

The enzyme-linked immunosorbent assay (ELISA) is the traditional method of accurately quantifying cytokine levels. This can be performed with high sensitivity and specificity. However, it is limited by the fact that only one cytokine can be measured at a time [10]. However, protein microarrays have recently evolved as a promising tool for quantifying proteins in biological samples [11, 12]. A protein microarray is, in many ways, a miniaturised version of a sandwich ELISA. Quantifying cytokine abundance by microarray typically involves printing capture antibody onto a coated glass slide and incubating with a serum sample. As with sandwich ELISA, target specificity is maximized through the use of paired sets of capture and detection antibodies, which bind to the same antigen via separate epitopes. In contrast to ELISAs, microarrays have the ability to evaluate multiple cytokines simultaneously. They require smaller sample volumes than ELISAs, are more sensitive, and have a greater dynamic range [13]. These factors make microarrays a cheaper and potentially favourable alternative to ELISAs for the large-scale detection of known proteins (reviewed in [14]).

In order to utilise microarray technology, factors such as slide surface; printing settings; print, wash, and blocking buffers; and detection methods must be optimised. In addition, multiplexing requires a further optimization above and beyond that performed for singleplex ELISAs and that of testing for cross-reactivity between the different capture and detection antibodies, reporter, and antigen targets. This is typically performed in a checkerboard or matrix experiment where each combination is tested as a monoplex of a specific antigen processed against the entire panel of capture antibodies and all detection antibodies. Following this, results must be shown to be reproducible and be validated against the established “gold standard,” the ELISA. As there are currently no guidelines for protein microarray biomarker assay development, reproducibility standards for this work were based on the Food and Drug Administration (FDA) guidelines for pharmacokinetic assay validation. These are 80–120% for accuracy and <20% coefficient of variation (CV) for precision [15].

In this study, the objective was to develop and validate a microarray platform to simultaneously quantify 16 biomarkers in the sera. The biomarkers were eotaxin-1, eotaxin-2, IFN-*γ*, IL-1*β*, IL-4, IL-6, IL-8, IL-10, IL-17, IL-23, IP-10, monocyte chemotactic protein-1 (MCP-1), receptor for advanced glycation end-products (RAGE), transforming growth factor-*β* (TGF-*β*), TNF-*α*, and vascular endothelial growth factor (VEGF). A direct comparison of ELISA and protein microarray was performed on 30 human sera as an integral part of the comprehensive validation of the microarray technique.

## 2. Methods

### 2.1. Serum Samples

The University of Nottingham Medical School Ethics Committee approved the study protocol and written informed consent was obtained from the 30 healthy participants before entering the study. Peripheral blood (10 mL) was collected in SST vacutainer tubes (BD); the serum was isolated and stored at −20°C.

### 2.2. Microarray Protocol

DuoSet paired antibody kits (R&D Systems, Minneapolis, MN) were used for the detection of 16 cytokines: eotaxin-1, eotaxin-2, IFN-*γ*, IL-1*β*, IL-4, IL-6, IL-8, IL-10, IL-17, IL-23, IP-10, MCP-1, RAGE, TGF-*β*, TNF-*α*, and VEGF. Each capture antibody diluted to 100 *μ*g/mL in PBS-Trehalose (Dulbeccos's phosphate buffered saline containing 50 mM D-(+) Trehalose) was loaded onto a 384-well plate (Genetix) and printed in in four sets of triplicate spots in a 16 × 16 array format onto a poly-l-lysine-coated glass slide (Thermofisher, UK) using a Biorobotics Microgrid II arrayer (Microgrid) and a silicon contact pin (Parallel Synthesis Technologies, USA). During printing, the array chamber was set to 60% humidity at 20°C, and the spot diameter was 315 *μ*m. Printed slides were left on the arrayer overnight, with the humidity turned off, and processed the next day. Parameters for the optimization of the protocol presented here are taken from Selvarajah, 2013 [16].

Slides were blocked with PBS-BSA (PBS containing 3% BSA; Sigma) for one hour and washed three times with PBS-Tween (PBs containing 0.05% Tween-20) wash buffer. A cocktail of recombinant protein standards was prepared containing each cytokine at a maximum recommended concentration for standard curve generation, according to manufacturers instructions (R&D Systems, Minneapolis, MN). The cocktail of standards was diluted twofold across eight dilutions and 50 *μ*L added to each block for 45 minutes. Slides were washed as above, and 50 *μ*L of an appropriately diluted cocktail of biotinylated detection antibodies, each at the manufacturers recommended concentration, was added to each block for 45 minutes. Following another wash, 50 *μ*L of 1 : 1000 diluted streptavidin-HRP (Opti-4CN amplification kit, #170-8238 Bio-Rad, USA) was added to each block and slides stored in the dark for 15 minutes. After further washing, 50 *μ*L of Bio-Rad amplification reagent (BAR, Opti-4CN amplification kit) was added to each block and slides were stored in the dark for 10 minutes. The slides were washed three times with PBS Tween containing 20% DMSO (v/v) and three times with wash buffer. Following this, 50 *μ*L of 1 : 1000 diluted streptavidin-conjugated Cy5 (final concentration 0.2 *μ*g/mL in wash buffer: #19-4317 E-Biosciences, UK) was added to each block and the slides were stored in the dark for 15 minutes. After a final wash, slides were rinsed in ultrapure water and dried by centrifugation. Slides were scanned at 635 nm and the data analysed using the GenePix Pro Software (Axon GenePix). Briefly, the median local background was subtracted from the median fluorescence of each spot and the corrected fluorescence was used to calculate the average fluorescence signal as well as the standard deviation. Unless stated otherwise, all incubation steps were performed at room temperature. The experiment was repeated at least twice for each biomarker.

To ascertain the accuracy of the microarray technique, two spike-in experiments were performed for each of the 16 biomarkers at three concentrations (22 pg/mL, 188 pg/mL, and 750 pg/mL). In the first experiment, recombinant protein standards for each of the 16 biomarkers at the above concentrations were spiked into PBS, while in the second experiment, the biomarkers were spiked into healthy donor serum, to identify if serum proteins had any detrimental effects on the assay system. Accuracy (%) was calculated based on the observed concentration measured against the expected concentration.

To determine the precision of the microarray platform, intra- and interassay comparisons were performed for all 16 biomarkers in the sera of up to 30 healthy volunteers. Intra-assay comparisons were performed sixteen times on the same slide simultaneously, while interassay comparisons were performed on different slides over a period of three consecutive days. The mean of the replicates and standard deviation were used to calculate the coefficients of variation (CV) of the microarray methodology. Significant differences between the variability of low-, mid-, and high-abundance cytokines were determined using the Kruskal-Wallis test and the post hoc Dunn's multiple comparison test.

### 2.3. ELISA Protocol

ELISAs were performed using R&D Systems DuoSet paired antibody kits (as per the microarray work) according to the manufacturer's instructions. Briefly, plates were coated with 1–4 *μ*g/mL of capture antibody and stored overnight. The plates were washed three times with 0.05% PBS-Tween (Sigma) and blocked with reagent diluent (1% BSA in PBS) for one hour. Following three washes as above, standards (prepared in reagent diluent in twofold dilutions, according to kit instructions) were added at 100 *μ*L per well in duplicate and the plate was incubated for two hours. The plate was washed and incubated with 100 *μ*L of appropriately diluted biotinylated detection antibody per well for a further two hours. After washing as above, 100 *μ*L of diluted streptavidin-HRP was added to each well and the plate was stored for 30 minutes in the dark. After a final wash, the plate was developed using the enzyme substrate peroxidase chromogen. After 30 minutes of incubation, the reaction was stopped by adding 50 *μ*L of 0.18 M H_2_SO_4_ per well and the absorbance read at 450 nm. All incubations steps were performed at room temperature.

## 3. Results

### 3.1. Microarray Validation

**Figure 1:**
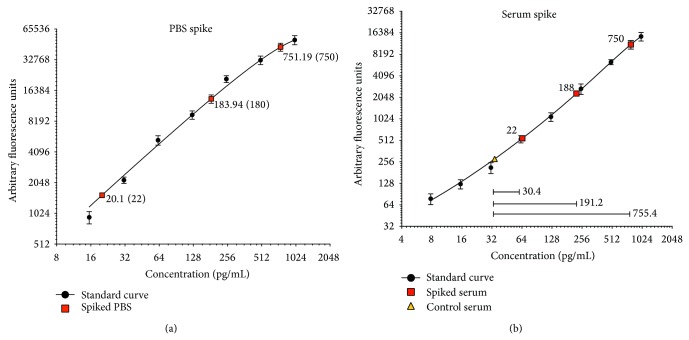
Microarray validation using spiked PBS and serum samples. (a) PBS was spiked with three known concentrations of protein: 22 pg/mL, 188 pg/mL, and 750 pg/mL. Standard curves were used to calculate the observed concentration in the PBS. Representative data for RAGE is shown. (b) The serum protein levels of biomarkers were quantified prior (triangle) to and after (squares) the addition of three known concentrations of cytokine (22 pg/mL, 188 pg/mL, and 750 pg/mL), by microarray. Standard curves were used to calculate the observed concentration differences between the control (nonspiked) and spiked serum samples (closed bars). The three inserted horizontal lines and numerical values in (b) indicate the difference between the quantified target level detected in control and spiked serum samples, that is, the difference due to the addition of the spike in each case. Representative data for VEGF is shown.

A microarray technique was developed to enable the quantification of 16 cytokine biomarkers in human sera. A series of validation tests were performed to ensure the reproducibility and accuracy of this technique. In the first instance, PBS (representative data for RAGE shown in Figure 1(a)) and serum (representative data for VEGF shown in Figure 1(b)) were spiked with three known concentrations of protein: 22 pg/mL, 188 pg/mL, and 750 pg/mL. The pre- and post-serum spike protein concentrations were quantified using a protein standard curve specific for each biomarker (Figure 1(b)). Serum was our sample of choice over plasma, due to the consistently lower background signal achieved with serum, relative to plasma for these microarrays (data not shown). BSA was our preferred blocking buffer for all experiments; 1% I-Block (purified casein) performs equally well (results not shown), but BSA is preferred due to cost and ease of preparation.

**Figure 2:**
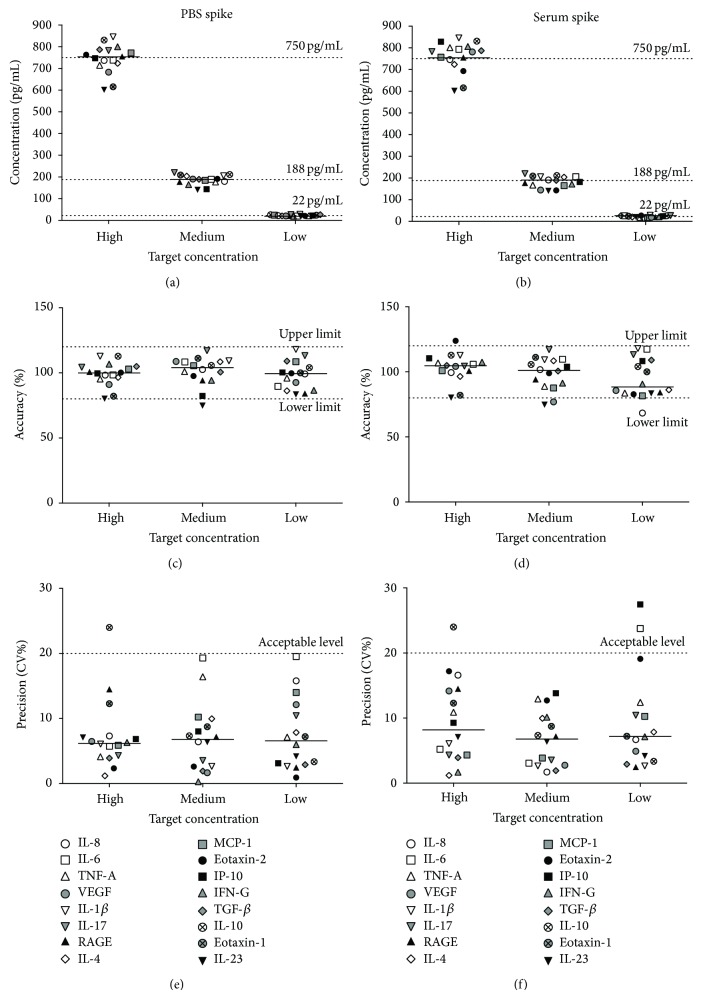
Microarray accuracy and precision of cytokine detection. PBS (a) and serum (b) spike experiments were performed for 16 cytokines at three known concentrations (22 pg/mL, 188 pg/mL, and 750 pg/mL). The expected (dashed lines) and observed concentration differences from these spiked experiments were determined by microarray. The accuracy of microarray cytokine detection was calculated for PBS (c) and serum (d) at each spike concentration. The majority of data fell within the acceptable limits of 80–120% accuracy, as outlined by the FDA [15]. The precision of microarray cytokine detection in PBS (e) and serum (f) spike experiments was also calculated. Again, the majority of data were within the limits of acceptability (<20% coefficient of variation, CV), as outlined by the FDA [15].

The accuracy and precision values of the microarray assay were then calculated from these data (Figure 2). The postspike observed values for the 16 biomarkers and expected values (dashed lines) of the biomarkers are shown in Figures 2(a) and 2(b). All but two of the high concentration spikes (750 pg/mL) were within 10% of the expected value, whereas all of the mid- and low-concentration spikes (22 pg/mL and 188 pg/mL) were within this range for the PBS spike (Figure 2(a)). The same was observed for the serum spike (Figure 2(b)). The observed mean protein values were within the acceptable criteria of accuracy (80–120%) for the vast majority of cytokines of the three spike concentrations (Figures 2(c) and 2(d)). The same was true for the acceptable level of precision (<20% CV, Figures 2(e) and 2(f)).

### 3.2. Microarray Limits of Detection and Quantification

The limits of sensitivity of the assay were measured using the lower limit of detection (LOD) and the lower and upper limits of quantification (LLOQ and ULOQ, resp.) [17]. The lower limit of detection (LOD) is the concentration of the biomarker required to give a signal equal to the background (blank) plus three times the standard deviation of the blank; the lower limit of quantification (LLOQ) is twice the level of the LOD or the point where the CV falls below 20%, whichever is highest. The lower limits of detection and quantification for the microarray assay are shown in Table 1. For the 16 biomarkers investigated, LOD values ranged from 0.284 to 1.9 pg/mL and LLOQ values ranged from 1.5 to 5.9 pg/mL. The upper limit of quantification (ULOQ) is the point at which the calculated precision does not exceed 15% of the CV and the accuracy is within 15% of the expected concentration [18] and this ranged from 750 to 1900 pg/mL for the 16 biomarkers tested (data not shown).

### 3.3. Microarray Intra- and Interassay Precision

**Figure 3:**
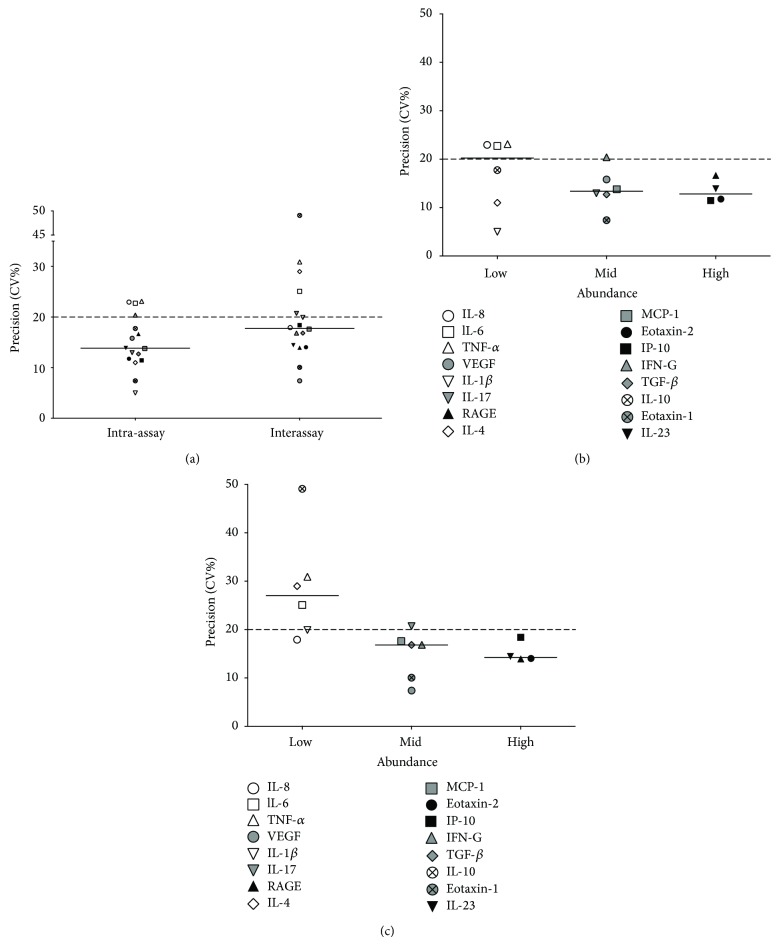
Microarray intra- and interassay precision. The intra- and interassay precision of microarray cytokine detection was calculated (a). Experiments to measure intra-assay precision were conducted on the same slide (*n* = 15 arrays), while those to measure interassay precision were performed on three consecutive days (*n* = 30 arrays per time point). The median precision values for intra- and interassay variation were within the acceptable limit of 20% coefficient of variation (CV), akin to the majority of individual cytokines. Cytokines were divided into low-abundance (<50 pg/mL), mid-abundance (51–199 pg/mL), and high-abundance (>200 pg/mL) groups for intra- and interassay variation ((b) and (c), resp.). All high-abundance cytokines, along with all but one mid-abundance cytokine, showed acceptable levels of intra- and interassay variation. The majority of low-abundance cytokines showed unacceptable interassay variation.

The microarray intra- and interassay variability, using the sera of up to 30 healthy volunteers, is shown in Figure 3. Three sets of two slides, containing 16 arrays per slide, were used for intra- and interassay variation testing. Identical samples were used on each set of two slides, at three independent time points. In the case of intra-assay variation, 13 of 16 biomarkers were within the acceptable limits of precision (<20% CV). When looking at interassay variation, 11 out of the 16 biomarkers were within the acceptable limits (Figure 3(a)).

The cytokine biomarkers were then subdivided into three groups, depending on their serum abundance, and each was analysed separately for intra- and interassay precision (Figures 3(b) and 3(c), resp.). Low-abundance cytokines were defined as those with a serum concentration of ≤50 pg/mL and were IL-1*β*, IL-4, IL-6, IL-8, IL-10, and TNF-*α*; mid-abundance cytokines, with a serum concentration of 51–199 pg/mL, were eotaxin-1, IL-17, IFN-*γ*, MCP-1, TGF-*β*, and VEGF; and high-abundance cytokines (serum concentration ≥200 pg/mL) were eotaxin-2, IL-23, IP-10, and RAGE. The precision values for 5 out of 6 mid-abundance cytokines and all four high-abundance cytokines were within the acceptable level of precision for both intra- and interassay variation. When looking at low-abundance cytokines, however, the median precision value for the intra-assay variation was on the limit of acceptable precision, with 3 cytokines outside the acceptable limit. As for the interassay variation, only one cytokine was within the acceptable level of precision and the median value for these low-abundance cytokines was outside the acceptable limits of precision.

### 3.4. Correlation between Microarray and ELISA Cytokine Detection Levels

**Figure 4:**
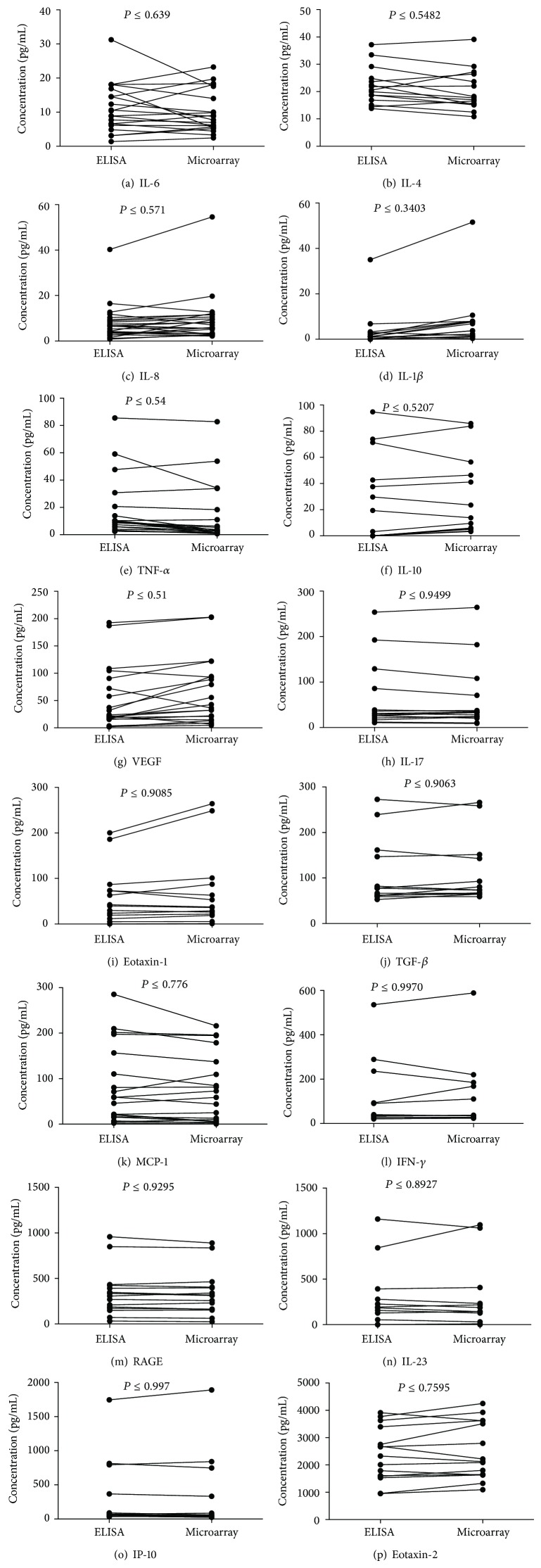
Correlation between microarray and ELISA cytokine detection levels. The levels of 16 cytokines were quantified by microarray and ELISA techniques in up to 30 human serum samples. Paired *t*-tests showed there were no significant differences between the two techniques for any of the cytokines tested. The 16 cytokines were eotaxin-1, eotaxin-2, IFN-*γ*, IL-1*β*, IL-4, IL-6, IL-8, IL-10, IL-17, IL-23, IP-10, MCP-1, RAGE, TGF-*β*, TNF-*α*, and VEGF.

**Figure 5:**
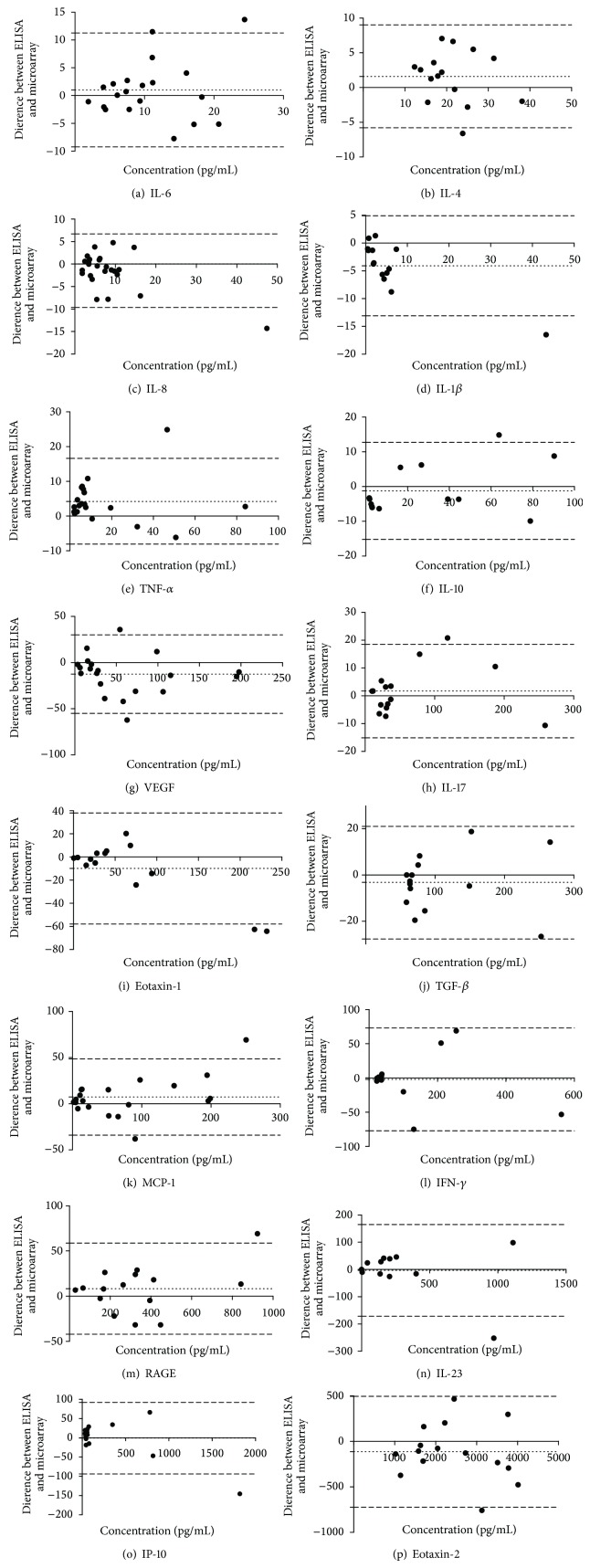
Bland Altman plots to compare ELISA and microarray. The acceptable level of bias is dictated by the thin dotted line on either side of zero. The dashed lines represent the 95% limits of agreement as the mean difference (2 SD). The differences between ELISA and microarray estimations show a notable advantage of the microarray technique in sensitivity especially for the low-abundance cytokines, such as IL-1*β* and IL-6.

The serum levels of the 16 cytokine biomarkers were quantified from 30 healthy individuals using both ELISA and the in-house microarray system; and the two detection methods were compared using paired *t*-tests (Figure 4) and visually represented in Bland-Altman plots (Figure 5). There were no significant differences in the level of cytokines detected by the two assays, for any of the 16 cytokines (*P* values ranged from 0.3404 to 0.997). This highlights the agreement between the two detection methods; in concordance with this, bias in favour of either technique was negligible (Figure 5). Furthermore, for less-abundant cytokines, such as IL-1*β*, the microarray platform appeared to be slightly more sensitive than the ELISA.

## 4. Discussion

Biomarkers can be very helpful in the diagnosis of disease, aid in the prognosis of disease, and/or help to predict treatment outcome. As a result, efforts to identify and quantify biomarkers have increased substantially in recent years. However, access to commercial protein microarray platforms is expensive and makes them unattainable for most [19]. Further adding to the problem is a lack of comprehensive guidelines for microarray protein biomarker validation studies. In this study, we validated our in-house sandwich microarray according to the FDA guidelines for pharmacokinetic assays.

Microarray spike recovery tests, performed on both PBS and human serum, showed accuracy and precision values of cytokine detection that were within the acceptable limits: 80 to 120% accuracy and <20% CV precision. This was in accordance with published literature [20–24]. Additionally, microarray intra- and interassay precision values were deemed acceptable for nine out of ten mid- and high-abundance cytokines, with the tenth being on the borderline of acceptability. As might be expected, there was a greater level of interassay variation compared to intra-assay variation and in low-abundance cytokines, such as IL-10, compared to mid- and high-abundance cytokines.

The limits of quantification are defined as the lowest and highest points in an assay that can be detected and quantified to an acceptable level of accuracy and precision. The microarray platform proved to be superior in sensitivity and has the advantage of detecting the lowest concentrations of cytokines [25, 26]. Whilst the LOD shown here is very low (less than 1 pg/mL for most of the examined cytokines), often it is not chosen as the limit to measure proteins in biological samples, as at this concentration there is a higher degree of variability between samples and thus the LLOQ is preferred when using microarray technology as the lowest analyte concentration that can be quantified to an acceptable level with both accuracy and precision.

Decisively, no significant differences were found between serum cytokine levels detected by microarray and the ELISA, the “gold standard” in immunoassays [27, 28], showing the two techniques to be comparable. However, for some low-abundance biomarkers, such as IL-1*β* and IL-6, there was less agreement between techniques. Interestingly, this may be due to the greater sensitivity of the microarray detection format [26, 29]; microarray fluorescence detection is measured over a range of many 10,000 s of fluorescence arbitrary units, compared to the discrete 0 to 3 optical density scale available in ELISAs.

One of our aims within this work was to produce a cost-effective method to support multiple cytokine/chemokine assays for large numbers of samples. With this in mind we have estimated the cost of performing equivalent 16 cytokine assays on multiple samples, assuming best use of reagents for commercial kits, and encompassing the number of plates/slides and associated secondary reagents required, but not pipette tips or technical time. Based upon this, we estimate that R&D Duoset kits would allow testing of 712 samples for each cytokine, in duplicate, requiring 240, 96-well plates and cost approximately £0.63 per cytokine per sample (£10 per sample for 16 cytokines).

Based upon similar best use of the same ELISA kits, our system would allow the testing of 2872 samples, with 4 technical replicates for each cytokine per array. Including slide, amplification and fluorescent dye costs equate to £0.18 per cytokine tested, or £2.88 per sample. This 71% saving comes with added technical replication, improved quality control based upon the analysis of spot morphology within the arrays, and reduced logistical issues due to the reduced number of plate “equivalent” assays required (roughly 1∖10th of the number of ELISA plates are required).

The closest equivalent commercial cytokine array is a 96-well plate array from Quantsys (testing 16 cytokines), which costs £11.45 per sample, or £0.71 per cytokine tested. Once again our system represents a 75% saving.

## 5. Conclusions

In summary, a series of experiments have been conducted to test the performance of an in-house microarray system in terms of accuracy, precision, and reproducibility. We have shown that 11 of the 16 cytokine biomarkers tested are in line with the FDA guidelines for analytical method validation. However, we saw a loss of precision at the lowest concentrations of protein in ra. This highlights the requirement for more detailed guidelines on acceptable levels of accuracy and precision, specifically with regard to biomarker detection, which are standardized across research institutions [30, 31]. This microarray system clearly offers great scope for the detection of biomarkers using microarrays. The in-house microarray protocol established here enables multiple biomarkers to be quantified simultaneously with relative ease, uses a smaller sample volume and is more sensitive than ELISA, and has lower running costs. Importantly, detection is not limited to the 16 biomarkers shown here but can be expanded as required.

## Figures and Tables

**Table 1:** Microarray lower limits of detection and quantification.

Biomarker	Lower limit of detection (LOD) (pg/mL)	Lower limit of quantification (LLOQ) (pg/mL)
Eotaxin-1	1.863	2.9
Eotaxin-2	0.739	5.9
IFN-*γ*	0.108	2.9
IL-1*β*	0.404	2.9
IL-4	0.600	2.9
IL-6	0.683	1.5
IL-8	0.535	1.5
IL-10	0.503	2.9
IL-17	0.445	5.9
IL-23	0.612	5.9
IP-10	0.458	5.9
MCP-1	0.284	5.9
RAGE	0.968	5.9
TGF-*β*	0.398	2.9
TNF-*α*	1.126	1.5
VEGF	0.744	1.5

The lower limit of detection (LOD) is the concentration of biomarker required to give a signal that is equal to the background (blank) plus three times the standard deviation of the blank. The lower limit of quantification (LLOQ) is twice the LOD value or the point where the coefficient of variation (CV), a measure of precision, falls below 20%, whichever is highest [17].

## Abbreviations

BSA: Bovine serum albumin
CV: Coefficient of variation
ELISA: Enzyme-linked immunosorbent assay
FDA: Food and Drug Administration
HRP: Horseradish peroxidase
IFN-*γ*: Interferon-gamma
IL: Interleukin
IP-10: Interferon-gamma-induced protein-10
LLOQ: Lower limit of quantification
LOD: Lower limit of detection
MCP-1: Monocyte chemotactic protein-1
PBS: Phosphate buffered saline
RAGE: Receptor for advanced glycation end-products
TGF-*β*: Transforming growth factor-beta
TNF-*α*: Tumour necrosis factor-alpha
ULOQ: Upper limit of quantification
VEGF: Vascular endothelial growth factor.
